# *catena*-Poly[[(aqualithium)-μ-3-carboxypyrazine-2-carboxylato-κ^4^ *O* ^2^,*N* ^1^:*O* ^3^,*N* ^4^] monohydrate]

**DOI:** 10.1107/S160053681102887X

**Published:** 2011-07-23

**Authors:** Wojciech Starosta, Janusz Leciejewicz

**Affiliations:** a Institute of Nuclear Chemistry and Technology, ul.Dorodna 16, 03-195 Warszawa, Poland

## Abstract

The polymeric structure of the title compound {[Li(C_6_H_3_N_2_O_4_)(H_2_O)]·H_2_O}_*n*_, contains two symmetry-independent Li^I^ complex units, both having distorted trigonal–bipyramidal coordination environments. The Li^I^ ions are bridged by both the N and O atoms of the ligands, generating two symmetry-independent polymeric chains propagating along the *b*-axis direction. In both ligands, the second carboxyl­ato O atom remains protonated, serving as a donor in a short intra­molecular O—H⋯O hydrogen bond. The coordination of each Li^I^ ion is completed by a water O atom. The ribbons are held together by a network of O—H⋯O hydrogen bonds in which the coordinated and uncoordinated water mol­ecules are donors and the carboxyl­ato O atoms act as acceptors.

## Related literature

For the crystal structures of two Li^I^ complexes with a pyrazine-2,3-dicarboxyl­ate ligand, see: Tombul *et al.* (2008 ▶); Tombul & Güven (2009 ▶). For the crystal structure of a Li^I^ complex with a pyrazine-2,3,5,6-tetra­carboxyl­ate ligand, see: Starosta & Leciejewicz (2010 ▶) and a pyrazine-2,5-dicarboxyl­ate ligand, see: Starosta & Leciejewicz (2011 ▶). For the crystal structures of two structural forms of pyrazine-2,3-dicarb­oxy­lic acid dihydrate, see: Takusagawa & Shimada (1973 ▶); Premkumar *et al.* (2004 ▶). For the crystal structures of Zn complexes with a pyrazine-2,3-dicarboxyl­ate ligand, see: Richard *et al.* (1974 ▶); Ptasiewicz-Bąk & Leciejewicz (1999 ▶); Gryz *et al.* (2005 ▶). 
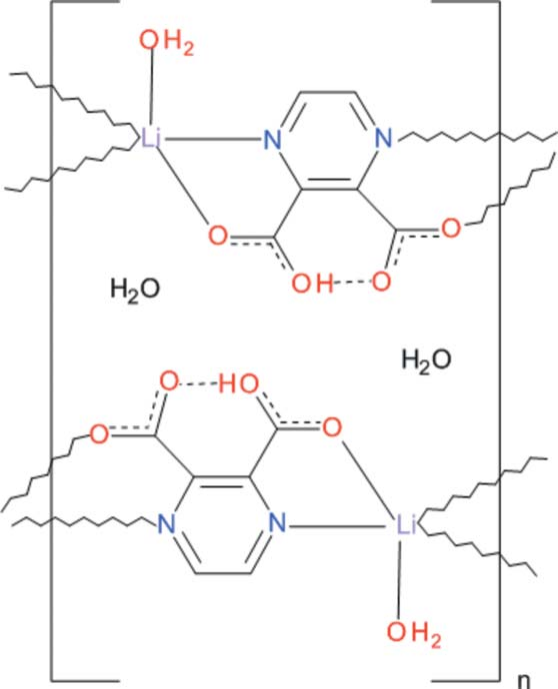


## Experimental

### 

#### Crystal data

[Li(C_6_H_3_N_2_O_4_)(H_2_O)]·H_2_O
*M* *_r_* = 210.08
Monoclinic, 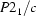

*a* = 12.673 (3) Å
*b* = 13.816 (3) Å
*c* = 10.956 (2) Å
β = 114.04 (3)°
*V* = 1752.0 (6) Å^3^
*Z* = 8
Mo *K*α radiation
μ = 0.14 mm^−1^
*T* = 293 K
0.32 × 0.14 × 0.09 mm

#### Data collection

Kuma KM4 four-circle diffractometer
Absorption correction: analytical (*CrysAlis RED*; Oxford Diffraction, 2008 ▶) *T*_min_ = 0.978, *T*_max_ = 0.984
3775 measured reflections
3595 independent reflections
1811 reflections with *I* > 2σ(*I*)
*R*_int_ = 0.064
3 standard reflections every 200 reflections  intensity decay: 3.4%

#### Refinement

*R*[*F* ^2^ > 2σ(*F* ^2^)] = 0.046
*wR*(*F* ^2^) = 0.149
*S* = 0.94
3595 reflections
311 parameters
H atoms treated by a mixture of independent and constrained refinement
Δρ_max_ = 0.28 e Å^−3^
Δρ_min_ = −0.36 e Å^−3^

### 

Data collection: *KM-4 Software* (Kuma, 1996 ▶); cell refinement: *KM-4 Software*; data reduction: *DATAPROC* (Kuma, 2001 ▶); program(s) used to solve structure: *SHELXS97* (Sheldrick, 2008 ▶); program(s) used to refine structure: *SHELXL97* (Sheldrick, 2008 ▶); molecular graphics: *SHELXTL* (Sheldrick, 2008 ▶); software used to prepare material for publication: *SHELXTL*.

## Supplementary Material

Crystal structure: contains datablock(s) I, global. DOI: 10.1107/S160053681102887X/kp2344sup1.cif

Structure factors: contains datablock(s) I. DOI: 10.1107/S160053681102887X/kp2344Isup2.hkl

Additional supplementary materials:  crystallographic information; 3D view; checkCIF report

## Figures and Tables

**Table 1:** Selected bond lengths (Å)

Li1—O11	2.014 (5)
Li1—N11	2.117 (4)
Li1—O15	1.985 (6)
Li1—O14^i^	2.005 (5)
Li1—N12^i^	2.162 (4)
Li2—O21	2.003 (5)
Li2—N21	2.138 (4)
Li2—O25	2.016 (5)
Li2—O24^ii^	2.024 (5)
Li2—N22^ii^	2.162 (4)

Symmetry codes: (i) 
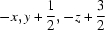
; (ii) 
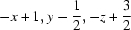
.

**Table 2:** Hydrogen-bond geometry (Å, °)

*D*—H⋯*A*	*D*—H	H⋯*A*	*D*⋯*A*	*D*—H⋯*A*
O2—H3⋯O24^ii^	0.83 (3)	2.12 (4)	2.943 (3)	170 (3)
O2—H4⋯O13^iii^	0.93 (4)	1.93 (4)	2.841 (3)	169 (3)
O25—H251⋯O1^ii^	0.86 (3)	1.83 (4)	2.640 (3)	155 (3)
O25—H252⋯O21^iv^	0.87 (4)	1.97 (4)	2.819 (3)	166 (3)
O15—H152⋯O25^v^	0.97 (4)	1.93 (4)	2.837 (3)	154 (3)
O15—H151⋯O2^vi^	0.72 (6)	2.11 (6)	2.816 (3)	165 (7)
O12—H131⋯O13	0.78 (6)	1.64 (6)	2.393 (3)	162 (7)
O23—H231⋯O22	0.67 (6)	1.76 (6)	2.416 (3)	170 (7)
O1—H1⋯O11	0.82 (3)	1.90 (3)	2.717 (3)	176 (3)
O1—H2⋯O22^vii^	0.91 (4)	1.90 (4)	2.808 (3)	174 (3)

Symmetry codes: (ii) 
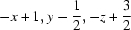
; (iii) 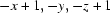; (iv) 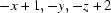; (v) 
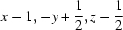
; (vi) 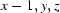; (vii) 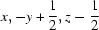.

**Fig. 1.**
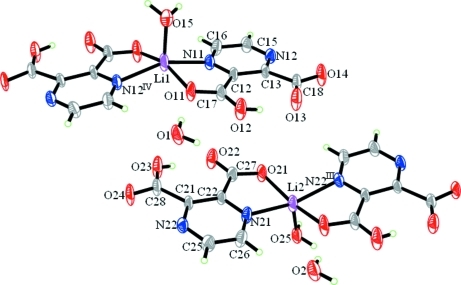
Two structural units of the title compound with atom labelling scheme and 50% probability displacement ellipsoids. Symmetry code: (i) -x, y - 1/2,-z + 3/2; (ii) -x + 1, y + 1/2, -z + 3/2; (iii) -x + 1, y - 1/2, -z + 3/2; (iv) -x, y + 1/2, -z + 3/2.

**Fig. 2.**
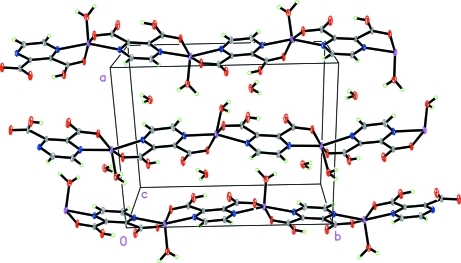
Packing diagram of the structure viewed along the b axis.

## supplementary crystallographic information

### Comment

Pyrazine-2,3-dicarboxylate ligand shows a marked tendency to form metal coordination compounds depending on the conditions of chemical preparation. Three Zn^II^ coordination polymers with the title ligand (2,3-PZDC) showing polymeric structures are known: a triclinic complex Zn(2,3-PZDC)(H_2_O)_2_.H_2_O (Richard *et al.*, 1974); a monoclinic complex Zn(2,3-PZDC)(H_2_O)_3_. H_2_O (Ptasiewicz-Bąk & Leciejewicz, 1999), and a monoclinic [(H_3_O)^1+^]_2_ [Zn(2,3-PZDC)_2_]^2-^ (Gryz, *et al.*, 2005). Two Li^I^ complexes with the title ligand have been recently reported (Tombul *et al.*, 2008; Tombul & Güven, 2009). The asymmetric unit of the title compound contains two symmetry independent Li^I^ ions, two ligand molecules, two coordinated and two crystal water molecules (Fig. 1, Table 1). Li^I^ ions and the ligands form two parallel molecular chains propagating in the *b* direction. In each, the Li ion shows a distorted trigonal bipyramidal coordination. The Li1 ion is 0.017 (1)Å out of the basal plane composed of carboxylato O11, O14^i^ and aqua O15 atoms; hetero N11 and N12^i^ atoms are at axial positions. The equatorial plane in the case of the Li2 ion consists of carboxylate O21, O24^ii^ and water O25 atoms; hetero N21 and N22^ii^ form the apices. The Li2 ion is 0.012 (1)Å out of the basal plane. The observed Li—O and Li—N bond distances are characteristic for Li^I^ complexes with diazine carboxylate ligands (Table 1). Each ligand uses both its N,*O* chelating sites to bridge Li^I^ ions. The second carboxylato O atoms do not participate in coordination but remain protonated to maintain the charge balance. In both ligands these protons are active in short intra-molecular hydrogen bonds of 2.393 (3) Å and 2.416 (3) Å (Table 2). The same effect has been also observed in another Li^I^ complex with the title ligand (Tombul *et al.*, 2008), a complex with pyrazine-2,3,5,6-tetracarboxylate ligand (Starosta & Leciejewicz, 2010) and a complex with pyrazine -2,5-dicarboxylate ligand (Starosta & Leciejewicz, 2011). Bond lengths and bond angles within both pyrazine rings do not differ from those reported in the structures of two modifications of the parent acid (Takusagawa & Shimada, 1973; Premkumar, *et al.*, 2004). Pyrazine rings are planar with r.m.s of 0.0040 (2) and 0.0094 (2) Å, for ligand 1 and 2, respectively. The carboxylate groups C17/O11/O12 and C8/O13/O14 make with the pyrazine ring 1 the dihedral angles of 4.8 (1)° and 3.8 (1)°, respectively. Dihedral angles made by the carboxylate groups C27/O21/O22 and C28/O23/O24 with the pyrazine ring 2 are 15.4 (2)° and 4.3 (1)°, respectively. Crystal and coordinated water molecules participate in a network of hydrogen bonds which connect the ribbons (Table 2, Fig. 2). They act as donors, the carboxylato O atoms as acceptors.

### Experimental

A solution of 2 mmol s of LiOH in 50 mL of doubly distilled cold water was titrated with an aqueous solution of pyrazine-2,3-dicarboxylic acid dihydrate until the pH reached the value of 5.5. Then, the solution was boiled under reflux with stirring for 6 h. After cooling to room temperature the solution was left to crystallise. Polycrystalline material which was found after evaporation to dryness was repeatedly recrystallised from cold water until single-crystal plates appeared. They were washed with methanol and dried in air.

### Refinement

Water hydrogen atoms were located  in a difference map and refined isotropically. H atoms attached to pyrazine-ring C atoms were positioned at calculated positions and were treated as riding on the parent atoms with C-H=0.93\%A and U*U*_ISO_(H)=1.2*U*_ISO_(C).

### Crystal data

**Table:**

[Li(C_6_H_3_N_2_O_4_)(H_2_O)]·H_2_O	*F*(000) = 864
*M**_r_* = 210.08	*D*_x_ = 1.593 Mg m^−^^3^
Monoclinic, *P*2_1_/*c*	Mo *K*α radiation, λ = 0.71073 Å
Hall symbol: -P 2ybc	Cell parameters from 25 reflections
*a* = 12.673 (3) Å	θ = 6–15°
*b* = 13.816 (3) Å	µ = 0.14 mm^−^^1^
*c* = 10.956 (2) Å	*T* = 293 K
β = 114.04 (3)°	Plates, colourless
*V* = 1752.0 (6) Å^3^	0.32 × 0.14 × 0.09 mm
*Z* = 8	

### Data collection

**Table:**

Kuma KM4 four-circle diffractometer	1811 reflections with *I* > 2σ(*I*)
Radiation source: fine-focus sealed tube	*R*_int_ = 0.064
graphite	θ_max_ = 27.7°, θ_min_ = 1.8°
profile data from ω/2θ scans	*h* = 0→14
Absorption correction: analytical (*CrysAlis RED*; Oxford Diffraction, 2008)	*k* = −18→0
*T*_min_ = 0.978, *T*_max_ = 0.984	*l* = −13→13
3775 measured reflections	3 standard reflections every 200 reflections
3595 independent reflections	intensity decay: 3.4%

### Refinement

**Table:**

Refinement on *F*^2^	Primary atom site location: structure-invariant direct methods
Least-squares matrix: full	Secondary atom site location: difference Fourier map
*R*[*F*^2^ > 2σ(*F*^2^)] = 0.046	Hydrogen site location: inferred from neighbouring sites
*wR*(*F*^2^) = 0.149	H atoms treated by a mixture of independent and constrained refinement
*S* = 0.94	*w* = 1/[σ^2^(*F*_o_^2^) + (0.0987*P*)^2^] where *P* = (*F*_o_^2^ + 2*F*_c_^2^)/3
3595 reflections	(Δ/σ)_max_ < 0.001
311 parameters	Δρ_max_ = 0.28 e Å^−^^3^
0 restraints	Δρ_min_ = −0.36 e Å^−^^3^

### Special details

**Table:**

Geometry. All e.s.d.'s (except the e.s.d. in the dihedral angle between two l.s. planes) are estimated using the full covariance matrix. The cell e.s.d.'s are taken into account individually in the estimation of e.s.d.'s in distances, angles and torsion angles; correlations between e.s.d.'s in cell parameters are only used when they are defined by crystal symmetry. An approximate (isotropic) treatment of cell e.s.d.'s is used for estimating e.s.d.'s involving l.s. planes.
Refinement. Refinement of *F*^2^ against ALL reflections. The weighted *R*-factor *wR* and goodness of fit *S* are based on *F*^2^, conventional *R*-factors *R* are based on *F*, with *F* set to zero for negative *F*^2^. The threshold expression of *F*^2^ > σ(*F*^2^) is used only for calculating *R*-factors(gt) *etc*. and is not relevant to the choice of reflections for refinement. *R*-factors based on *F*^2^ are statistically about twice as large as those based on *F*, and *R*- factors based on ALL data will be even larger.

### Fractional atomic coordinates and isotropic or equivalent isotropic displacement parameters (Å^2^)

**Table:**

	*x*	*y*	*z*	*U*_iso_*/*U*_eq_	
N11	−0.01503 (17)	0.17705 (13)	0.7604 (2)	0.0323 (5)	
N12	0.00129 (18)	−0.02194 (13)	0.7752 (2)	0.0325 (5)	
O14	0.1043 (2)	−0.12689 (12)	0.6604 (2)	0.0553 (6)	
C13	0.0533 (2)	0.02779 (15)	0.7099 (2)	0.0283 (5)	
O13	0.17126 (19)	−0.00377 (13)	0.5878 (2)	0.0544 (6)	
O11	0.07742 (16)	0.28612 (11)	0.6413 (2)	0.0462 (5)	
O12	0.1688 (2)	0.16941 (14)	0.5894 (2)	0.0540 (6)	
C17	0.1009 (2)	0.19946 (16)	0.6398 (3)	0.0351 (6)	
C12	0.0454 (2)	0.12986 (15)	0.7036 (2)	0.0282 (5)	
C18	0.1147 (2)	−0.03926 (17)	0.6484 (3)	0.0354 (6)	
C15	−0.0584 (2)	0.02637 (18)	0.8300 (3)	0.0376 (6)	
H15	−0.0953	−0.0075	0.8747	0.045*	
C16	−0.0671 (2)	0.12673 (18)	0.8224 (3)	0.0372 (6)	
H16	−0.1101	0.1588	0.8614	0.045*	
O21	0.41371 (16)	0.10101 (11)	0.8670 (2)	0.0406 (5)	
N22	0.51761 (17)	0.40335 (13)	0.7446 (2)	0.0318 (5)	
O24	0.39022 (18)	0.51517 (11)	0.8178 (2)	0.0496 (6)	
O22	0.33218 (18)	0.22600 (12)	0.9203 (2)	0.0523 (6)	
N21	0.53363 (18)	0.20579 (13)	0.7753 (2)	0.0345 (5)	
O23	0.3238 (2)	0.40041 (15)	0.9029 (3)	0.0603 (7)	
C28	0.3850 (2)	0.42871 (16)	0.8427 (3)	0.0374 (6)	
C22	0.4624 (2)	0.25575 (15)	0.8148 (2)	0.0271 (5)	
C23	0.4556 (2)	0.35762 (14)	0.8001 (2)	0.0271 (5)	
C27	0.3966 (2)	0.18992 (16)	0.8712 (3)	0.0324 (6)	
C26	0.5974 (2)	0.25337 (17)	0.7252 (3)	0.0404 (7)	
H26	0.6494	0.2196	0.7009	0.048*	
C25	0.5878 (2)	0.35298 (17)	0.7085 (3)	0.0383 (6)	
H25	0.6321	0.3847	0.6708	0.046*	
Li2	0.5322 (4)	0.0529 (3)	0.8042 (4)	0.0371 (10)	
O25	0.66173 (18)	0.02579 (12)	0.9834 (2)	0.0389 (5)	
O1	0.2117 (2)	0.38123 (13)	0.5402 (2)	0.0502 (6)	
O15	−0.1887 (2)	0.31162 (16)	0.5473 (2)	0.0532 (6)	
Li1	−0.0422 (4)	0.3254 (3)	0.7089 (5)	0.0416 (10)	
O2	0.71760 (19)	0.12508 (13)	0.5331 (2)	0.0459 (5)	
H3	0.695 (3)	0.092 (2)	0.581 (3)	0.043 (9)*	
H4	0.754 (3)	0.092 (3)	0.488 (4)	0.080 (12)*	
H251	0.707 (3)	−0.009 (2)	0.961 (3)	0.055 (9)*	
H252	0.633 (3)	−0.004 (3)	1.033 (3)	0.064 (11)*	
H152	−0.252 (3)	0.355 (3)	0.538 (4)	0.083 (12)*	
H151	−0.223 (5)	0.268 (5)	0.534 (7)	0.15 (3)*	
H131	0.183 (5)	0.115 (4)	0.590 (6)	0.15 (2)*	
H231	0.329 (5)	0.353 (4)	0.916 (5)	0.12 (2)*	
H1	0.169 (2)	0.354 (2)	0.568 (3)	0.032 (8)*	
H2	0.248 (3)	0.342 (3)	0.501 (4)	0.079 (12)*	

### Atomic displacement parameters (Å^2^)

**Table:**

	*U*^11^	*U*^22^	*U*^33^	*U*^12^	*U*^13^	*U*^23^
N11	0.0411 (12)	0.0160 (9)	0.0441 (12)	0.0021 (8)	0.0218 (10)	0.0018 (8)
N12	0.0398 (11)	0.0159 (9)	0.0466 (13)	0.0001 (8)	0.0227 (10)	0.0039 (8)
O14	0.0881 (15)	0.0120 (8)	0.0887 (16)	−0.0007 (9)	0.0594 (13)	−0.0029 (9)
C13	0.0363 (13)	0.0097 (10)	0.0397 (13)	0.0019 (9)	0.0163 (11)	0.0003 (9)
O13	0.0865 (15)	0.0181 (9)	0.0901 (16)	0.0039 (9)	0.0680 (14)	0.0005 (9)
O11	0.0621 (12)	0.0096 (8)	0.0848 (15)	0.0024 (8)	0.0482 (12)	0.0064 (8)
O12	0.0775 (15)	0.0164 (9)	0.0968 (17)	0.0029 (9)	0.0648 (14)	0.0060 (10)
C17	0.0456 (14)	0.0131 (10)	0.0541 (17)	0.0001 (10)	0.0279 (13)	0.0036 (10)
C12	0.0354 (13)	0.0127 (10)	0.0380 (13)	0.0035 (9)	0.0164 (11)	0.0023 (9)
C18	0.0506 (16)	0.0155 (11)	0.0486 (15)	0.0026 (10)	0.0288 (13)	−0.0020 (10)
C15	0.0490 (15)	0.0209 (12)	0.0519 (16)	0.0001 (11)	0.0298 (13)	0.0058 (11)
C16	0.0474 (15)	0.0234 (12)	0.0483 (15)	0.0073 (11)	0.0272 (13)	0.0054 (11)
O21	0.0552 (11)	0.0099 (7)	0.0693 (13)	0.0023 (7)	0.0383 (10)	0.0053 (7)
N22	0.0382 (12)	0.0162 (9)	0.0433 (12)	0.0031 (8)	0.0187 (10)	0.0052 (8)
O24	0.0805 (14)	0.0101 (8)	0.0791 (15)	0.0036 (8)	0.0538 (12)	0.0022 (8)
O22	0.0747 (14)	0.0180 (8)	0.0953 (16)	0.0062 (9)	0.0666 (13)	0.0091 (9)
N21	0.0464 (12)	0.0153 (9)	0.0504 (13)	0.0069 (9)	0.0286 (11)	0.0053 (9)
O23	0.0965 (19)	0.0157 (9)	0.1049 (19)	0.0075 (10)	0.0781 (16)	0.0063 (10)
C28	0.0554 (17)	0.0122 (10)	0.0566 (17)	0.0037 (10)	0.0352 (14)	0.0018 (10)
C22	0.0348 (13)	0.0119 (10)	0.0377 (14)	0.0031 (9)	0.0182 (11)	0.0038 (9)
C23	0.0343 (12)	0.0112 (10)	0.0388 (14)	0.0022 (9)	0.0179 (11)	0.0020 (9)
C27	0.0399 (14)	0.0161 (10)	0.0458 (15)	0.0021 (10)	0.0223 (12)	0.0058 (10)
C26	0.0515 (16)	0.0216 (12)	0.0630 (18)	0.0098 (11)	0.0386 (15)	0.0085 (11)
C25	0.0473 (15)	0.0238 (13)	0.0537 (17)	0.0024 (11)	0.0307 (14)	0.0100 (11)
Li2	0.052 (3)	0.0131 (17)	0.053 (3)	0.0028 (17)	0.028 (2)	0.0018 (17)
O25	0.0508 (12)	0.0159 (8)	0.0592 (13)	0.0004 (8)	0.0319 (10)	−0.0022 (8)
O1	0.0647 (14)	0.0193 (9)	0.0889 (17)	−0.0108 (9)	0.0540 (13)	−0.0094 (10)
O15	0.0506 (13)	0.0231 (10)	0.0787 (16)	0.0043 (10)	0.0190 (11)	0.0035 (10)
Li1	0.056 (3)	0.0126 (19)	0.065 (3)	0.0019 (17)	0.033 (2)	0.0019 (18)
O2	0.0672 (14)	0.0219 (9)	0.0581 (13)	−0.0051 (9)	0.0351 (12)	−0.0045 (9)

### Geometric parameters (Å, °)

**Table:**

N11—C16	1.321 (3)	O24—C28	1.233 (3)
N11—C12	1.337 (3)	Li2—O24^ii^	2.024 (5)
Li1—O11	2.014 (5)	O22—C27	1.250 (3)
Li1—N11	2.117 (4)	N21—C26	1.322 (3)
Li1—O15	1.985 (6)	N21—C22	1.340 (3)
Li1—O14^i^	2.005 (5)	O23—C28	1.267 (3)
Li1—N12^i^	2.162 (4)	O23—H231	0.67 (6)
N12—C15	1.322 (3)	C28—C23	1.524 (3)
N12—C13	1.342 (3)	C22—C23	1.415 (3)
Li1—N12^i^	2.162 (4)	C22—C27	1.525 (3)
O14—C18	1.231 (3)	C26—C25	1.387 (3)
Li1—O14^i^	2.005 (5)	C26—H26	0.9300
C13—C12	1.413 (3)	C25—H25	0.9300
C13—C18	1.531 (3)	Li2—O21	2.003 (5)
O13—C18	1.258 (3)	Li2—N21	2.138 (4)
O11—C17	1.235 (3)	Li2—O25	2.016 (5)
O12—C17	1.267 (3)	Li2—O24^ii^	2.024 (5)
O12—H131	0.78 (6)	Li2—N22^ii^	2.162 (4)
C17—C12	1.519 (3)	O25—H251	0.86 (3)
C15—C16	1.391 (3)	O25—H252	0.87 (4)
C15—H15	0.9300	O1—H1	0.82 (3)
C16—H16	0.9300	O1—H2	0.91 (4)
O21—C27	1.251 (3)	O15—H152	0.97 (4)
N22—C25	1.312 (3)	O15—H151	0.72 (6)
N22—C23	1.333 (3)	O2—H3	0.83 (3)
Li2—N22^ii^	2.162 (4)	O2—H4	0.93 (4)
			
C16—N11—C12	118.9 (2)	C23—C22—C27	128.4 (2)
C16—N11—Li1	125.8 (2)	N22—C23—C22	120.2 (2)
C12—N11—Li1	114.20 (19)	N22—C23—C28	111.30 (18)
C15—N12—C13	118.65 (19)	C22—C23—C28	128.5 (2)
C15—N12—Li1^iii^	128.2 (2)	O22—C27—O21	124.1 (2)
C13—N12—Li1^iii^	112.88 (19)	O22—C27—C22	119.8 (2)
C18—O14—Li1^iii^	119.3 (2)	O21—C27—C22	116.0 (2)
N12—C13—C12	119.8 (2)	N21—C26—C25	120.8 (2)
N12—C13—C18	111.86 (18)	N21—C26—H26	119.6
C12—C13—C18	128.4 (2)	C25—C26—H26	119.6
C17—O11—Li1	119.2 (2)	N22—C25—C26	121.3 (2)
C17—O12—H131	121 (5)	N22—C25—H25	119.3
O11—C17—O12	122.2 (2)	C26—C25—H25	119.3
O11—C17—C12	116.6 (2)	O21—Li2—O25	98.9 (2)
O12—C17—C12	121.1 (2)	O21—Li2—O24^ii^	161.1 (3)
N11—C12—C13	120.4 (2)	O25—Li2—O24^ii^	100.0 (2)
N11—C12—C17	111.46 (18)	O21—Li2—N21	77.02 (15)
C13—C12—C17	128.1 (2)	O25—Li2—N21	106.0 (2)
O14—C18—O13	123.3 (2)	O24^ii^—Li2—N21	96.94 (19)
O14—C18—C13	116.9 (2)	O21—Li2—N22^ii^	102.6 (2)
O13—C18—C13	119.8 (2)	O25—Li2—N22^ii^	95.94 (17)
N12—C15—C16	121.5 (2)	O24^ii^—Li2—N22^ii^	76.26 (15)
N12—C15—H15	119.3	N21—Li2—N22^ii^	157.9 (3)
C16—C15—H15	119.3	Li2—O25—H251	101 (2)
N11—C16—C15	120.7 (2)	Li2—O25—H252	108 (2)
N11—C16—H16	119.6	H251—O25—H252	114 (3)
C15—C16—H16	119.6	H1—O1—H2	116 (3)
C27—O21—Li2	120.3 (2)	Li1—O15—H152	118 (2)
C25—N22—C23	119.0 (2)	Li1—O15—H151	122 (5)
C25—N22—Li2^iv^	126.7 (2)	H152—O15—H151	96 (5)
C23—N22—Li2^iv^	113.48 (19)	O15—Li1—O14^i^	99.9 (2)
C28—O24—Li2^iv^	118.7 (2)	O15—Li1—O11	102.5 (2)
C26—N21—C22	118.94 (19)	O14^i^—Li1—O11	157.6 (3)
C26—N21—Li2	126.6 (2)	O15—Li1—N11	97.8 (2)
C22—N21—Li2	114.44 (19)	O14^i^—Li1—N11	101.3 (2)
C28—O23—H231	113 (5)	O11—Li1—N11	76.82 (16)
O24—C28—O23	120.9 (2)	O15—Li1—N12^i^	105.8 (2)
O24—C28—C23	117.7 (2)	O14^i^—Li1—N12^i^	76.87 (16)
O23—C28—C23	121.3 (2)	O11—Li1—N12^i^	95.81 (19)
N21—C22—C23	119.6 (2)	N11—Li1—N12^i^	156.3 (3)
N21—C22—C27	112.03 (19)	H3—O2—H4	116 (3)

Symmetry codes: (i) −*x*, *y*+1/2, −*z*+3/2; (ii) −*x*+1, *y*−1/2, −*z*+3/2; (iii) −*x*, *y*−1/2, −*z*+3/2; (iv) −*x*+1, *y*+1/2, −*z*+3/2.

### Hydrogen-bond geometry (Å, °)

**Table:**

*D*—H···*A*	*D*—H	H···*A*	*D*···*A*	*D*—H···*A*
O2—H3···O24^ii^	0.83 (3)	2.12 (4)	2.943 (3)	170 (3)
O2—H4···O13^v^	0.93 (4)	1.93 (4)	2.841 (3)	169 (3)
O25—H251···O1^ii^	0.86 (3)	1.83 (4)	2.640 (3)	155 (3)
O25—H252···O21^vi^	0.87 (4)	1.97 (4)	2.819 (3)	166 (3)
O15—H152···O25^vii^	0.97 (4)	1.93 (4)	2.837 (3)	154 (3)
O15—H151···O2^viii^	0.72 (6)	2.11 (6)	2.816 (3)	165 (7)
O12—H131···O13	0.78 (6)	1.64 (6)	2.393 (3)	162 (7)
O23—H231···O22	0.67 (6)	1.76 (6)	2.416 (3)	170 (7)
O1—H1···O11	0.82 (3)	1.90 (3)	2.717 (3)	176 (3)
O1—H2···O22^ix^	0.91 (4)	1.90 (4)	2.808 (3)	174 (3)

Symmetry codes: (ii) −*x*+1, *y*−1/2, −*z*+3/2; (v) −*x*+1, −*y*, −*z*+1; (vi) −*x*+1, −*y*, −*z*+2; (vii) *x*−1, −*y*+1/2, *z*−1/2; (viii) *x*−1, *y*, *z*; (ix) *x*, −*y*+1/2, *z*−1/2.
